# Pixel-Attention W-Shaped Network for Joint Lesion Segmentation and Diabetic Retinopathy Severity Staging

**DOI:** 10.3390/diagnostics15202619

**Published:** 2025-10-17

**Authors:** Archana Singh, Sushma Jain, Vinay Arora

**Affiliations:** Department of Computer Science & Engineering, Thapar Institute of Engineering & Technology, Patiala 147004, India; sjain@thapar.edu (S.J.); vinay.arora@thapar.edu (V.A.)

**Keywords:** pixel-attention, W-shaped network, lesion segmentation, diabetic retinopathy

## Abstract

**Background:** Visual impairment remains a critical public health challenge, and diabetic retinopathy (DR) is a leading cause of preventable blindness worldwide. Early stages of the disease are particularly difficult to identify, as lesions are subtle, expert review is time-consuming, and conventional diagnostic workflows remain subjective. **Methods:** To address these challenges, we propose a novel Pixel-Attention W-shaped (PAW-Net) deep learning framework that integrates a Lesion-Prior Cross Attention (LPCA) module with a W-shaped encoder–decoder architecture. The LPCA module enhances pixel-level representation of microaneurysms, hemorrhages, and exudates, while the dual-branch W-shaped design jointly performs lesion segmentation and disease severity grading in a single, clinically interpretable pass. The framework has been trained and validated using DDR and a preprocessed Messidor + EyePACS dataset, with APTOS-2019 reserved for external, out-of-distribution evaluation. **Results:** The proposed PAW-Net framework achieved robust performance across severity levels, with an accuracy of 98.65%, precision of 98.42%, recall (sensitivity) of 98.83%, specificity of 99.12%, F1-score of 98.61%, and a Dice coefficient of 98.61%. Comparative analyses demonstrate consistent improvements over contemporary architectures, particularly in accuracy and F1-score. **Conclusions:** The PAW-Net framework generates interpretable lesion overlays that facilitate rapid triage and follow-up, exhibits resilience under domain shift, and maintains an efficient computational footprint suitable for telemedicine and mobile deployment.

## 1. Introduction

**Figure 1:**
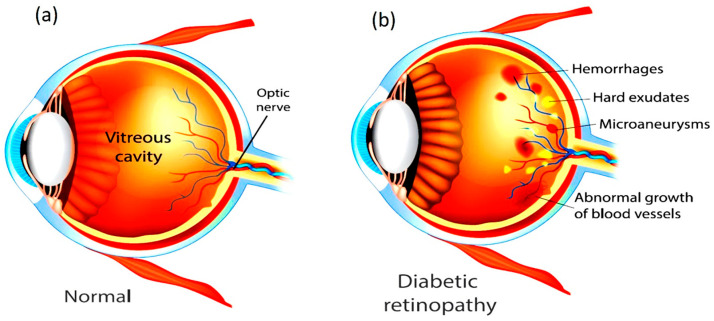
Eye images (**a**) Healthy eye and (**b**) Diabetic Retinopathy-affected Eye [4].

Diabetes mellitus is one of the most pressing global health challenges of the 21st century. According to the International Diabetes Federation, about 463 million people worldwide were affected by diabetes in 2019, and this figure is projected to rise to nearly 700 million by 2045 [1]. With this growing prevalence, diabetes-associated complications are also on the rise, among which DR is one of the most severe and vision-threatening outcomes. DR currently affects more than 400 million individuals across the globe and remains a leading cause of preventable blindness [2]. The disease develops due to persistent hyperglycemia that damages the retinal microvasculature, leading to swelling, leakage, and blockage of blood vessels in the retina, the light-sensitive tissue responsible for transmitting visual information to the brain. In its early stages, DR often progresses silently without noticeable symptoms, but as the disease advances, patients may experience blurred vision, altered light perception, and ultimately irreversible blindness if untreated [3]. Figure 1 shows the difference between a Healthy Eye and an Eye affected with DR. Both type 1 and type 2 diabetic patients are highly susceptible, particularly when glycemic control is inadequate. Clinically, the condition advances from Non-Proliferative Diabetic Retinopathy (NPDR), characterized by microaneurysms and hemorrhages, to Proliferative Diabetic Retinopathy (PDR), where pathological neovascularization can result in severe complications such as vitreous hemorrhage, retinal detachment, and secondary glaucoma. Beyond hyperglycemia, risk factors such as long disease duration, hypertension, dyslipidemia, smoking, and pregnancy further accelerate disease progression. DR is now also considered an inflammatory neurovascular disorder with neurological involvement, disproportionately affecting underserved regions where limited access to healthcare delays timely management [4].

**Figure 2:**
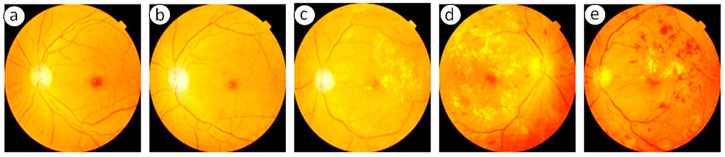
Fundus Images showing (**a**) No DR, (**b**) Mild, (**c**) Moderate, (**d**) Severe and (**e**) PDR [4].

Despite its severity, DR can be prevented or its progression slowed through proper disease management. Maintaining a healthy diet, engaging in regular physical activity, and adhering to effective diabetes care significantly reduce the likelihood of developing DR. Close monitoring of blood sugar levels, along with strict control of associated risk factors, is essential [5]. Depending on disease severity, treatment may involve pharmacological interventions, laser therapy, or surgical procedures. Regular eye examinations and early diagnosis play a critical role in minimizing the risk of vision loss. Collaboration between patients and healthcare providers is therefore vital in implementing effective strategies for prevention, timely intervention, and long-term management of DR. Advancements in science and technology have significantly improved human living standards by making daily life safer, simpler, and more convenient. Among these innovations, automated systems powered by Machine Learning (ML) have emerged as valuable tools for delivering a wide range of services aimed at improving quality of life [6]. In the healthcare domain, ML and Deep Learning (DL) methods have become particularly important for enabling accurate and early diagnosis of critical diseases. With the rapid increase in the number of diabetes patients, retinal screening programs must capture and analyze a growing volume of retinal images. To ensure consistency and reliability, automated systems are increasingly required to classify these images and provide severity assessments comparable to professional clinical evaluations. Widely adopted clinical standards for such assessments include the International Clinical Diabetic Retinopathy (ICDR) grading system and the diabetic macular edema disease severity scale. NPDR represents the early and less severe stage of DR, whereas PDR corresponds to the advanced and more vision-threatening form. In the initial stages of NPDR, patients may present with mild symptoms such as blurred vision, but as the disease progresses, abnormal neovascularization occurs in the retina, greatly increasing the risk of severe vision loss [7]. Chronic hyperglycemia in diabetes mellitus leads to vascular dysfunction, causing capillary dilation, leakage of fluids, and eventual retinal damage. These pathological changes manifest as characteristic lesions in fundus retinal images, which are broadly classified into red and bright lesions. Red lesions include microaneurysms (MA) and hemorrhages (HEM), appearing as small and large dark-red spots, respectively. Bright lesions consist of hard and soft exudates (EX) [8]. Hard exudates typically appear as sharply defined, bright yellow deposits, whereas soft exudates, also known as cotton-wool spots, are fluffy, yellowish-white patches associated with nerve fibre layer damage. For better clarity regarding the available fundus images and their categories, Figure 2 illustrates the different stages of DR, ranging from No DR to PDR. Accurate identification of these lesions is crucial for grading disease severity, as they serve as clinical biomarkers of progression from NPDR to PDR. Recent studies suggest that DL-based automated diagnostic systems show strong potential in reliably detecting such lesion patterns, thereby supporting early and accurate diagnosis of DR.

However, many existing DL models treat lesion segmentation and severity classification as separate tasks, leading to redundancy and reduced clinical interpretability. Moreover, conventional convolutional architectures struggle to capture long-range contextual relationships across retinal structures, which limits their ability to identify subtle lesion patterns during the early stages of DR. Although attention mechanisms have recently improved lesion localization, their integration into unified multi-branch frameworks for both lesion detection and staging is still limited. These shortcomings underline the necessity for a robust, clinically coherent solution. The proposed Pixel-Attention W-shaped Network (PAW-Net) addresses this need by combining a Lesion-Prior Cross Attention (LPCA) module with a dual-branch W-shaped encoder–decoder architecture. This design enhances pixel-level lesion representation while simultaneously enabling disease severity grading within a single, end-to-end model. Unlike prior methods, PAW-Net provides an interpretable, efficient, and clinically aligned approach, filling a crucial gap between lesion-focused analysis and reliable DR staging. The practical novelty of this study lies in its ability to unify these processes, thereby offering clinicians an accurate and comprehensive diagnostic tool for improving patient outcomes.

This paper is structured into five main sections to present the research in a logical and coherent manner. Section 1 defines the research problem, objectives, and relevance of the study, setting the background and motivation. Section 2 provides a critical analysis of prior studies, highlights existing knowledge gaps, and formulates the theoretical foundation for the present work. Section 3 details the experimental design, techniques, and procedures adopted in the investigation. Section 4 reports the experimental outcomes, interprets their significance, and situates them in comparison with earlier findings. Finally, Section 5 distils the core contributions of the research, emphasising its practical implications and suggesting directions for future exploration.

### Key Contributions

The main contributions of this study are summarized as follows:

• Lightweight Encoder–Decoder with Pixel Attention: A customized encoder–decoder architecture has been developed, integrating pixel-level attention mechanisms to achieve precise lesion segmentation and enhance sensitivity to subtle pathological features.
• Enhanced Segmentation and Classification Performance: The proposed framework has demonstrated superior accuracy compared to state-of-the-art methods in both lesion segmentation and image-level classification. Particular improvements have been observed in the detection of early-stage DR lesions and in mitigating the effects of class imbalance.
• Clinical Adaptability and Practical Deployment: The model has been validated for real-world clinical applicability, demonstrating potential for seamless integration into screening platforms and suitability for deployment in resource-constrained environments without compromising diagnostic reliability in telemedicine contexts.

These contributions have established the proposed framework as a robust, scalable, and clinically relevant tool for large-scale DR screening and early intervention.

## 2. Literature Review

In recent years, DR research has advanced from purely classification models to joint segmentation-classification frameworks that integrate lesion-level interpretation. These systems increasingly leverage attention mechanisms, lesion/vascular priors, multi-task design, and robust preprocessing, all of which align closely with PAW-Net’s core innovations. Li et al. (2024) proposed a multi-lesion segmentation-guided deep attention network that segments microaneurysms, hemorrhages, and exudates, then uses those lesion maps to inform DR severity grading [9]. Their work demonstrated that combining lesion segmentation with classification improves grading accuracy significantly, especially for moderate and severe stages.

Another study by Sharma et al. (2025) developed a hybrid model combining CNN feature extraction with genetic algorithms and SVM classification [10]. They focused on exudate/hemorrhage segmentation followed by classification, showing gains in accuracy and interpretability when lesion features are explicitly used as priors. In “Lesion classification and DR grading” (Liu et al., 2024), the authors emphasize that tiny lesions at early DR stages are especially difficult to detect and often confused with retinal vessels [11]. Their framework uses multi-scale attention and specialized preprocessing to enhance these subtle features prior to grading. Chandhakanond & Aimmanee (2025) used iterative NICK thresholding, watershed segmentation, and χ^2^ feature ranking to segment exudates and hemorrhages [12]. Their segmentation performance, though lower in harsh visual conditions, showed that traditional image-processing + feature selection methods remain competitive for lesion localization. Dinesen et al. (2025) targeted PDR more specifically and built a segmentation model for new retinal vessels and preretinal hemorrhages in six-field retinal images to distinguish active vs. inactive PDR [13]. This focuses on disease progression and aligns with PAW-Net’s capacity to highlight clinically urgent lesion types.

Bappi et al. (2025) provided a strong example of combining robust preprocessing, lesion awareness, and CNN-based classification, with clear improvements in detection and grading performance on large public datasets [14]. Their methods show how balancing segmentation and classification tasks can yield state-of-the-art results. Another relevant model, “DeepDR-LLM” (Li et al., 2024), uses a transformer-based architecture that includes image quality assessment, lesion segmentation, and DR/DME grading modules [15]. Their modular design supports both interpretability and adaptation to portable imaging settings, important for screening in resource-limited environments.

Harisha et al. (2024) explored DR detection using CNNs in the APTOS-2019 competition. They emphasized the challenge of grading consistency across image quality variations and stressed the need for preprocessing (illumination correction, trimming, artifact removal) to maintain segmentation accuracy [16].

Multi-task and multi-branch methods like MaMNet (Xing et al., 2023) show that splitting branches to focus separately on low-level lesion features and higher-level context improves performance for DR and DME grading [17]. While DME is beyond our immediate focus, its architectural style parallels PAW-Net’s dual-decoder/dual-branch approach. Finally, Dejene et al. (2025) conducted a comprehensive review of recent AI-based DR screening models, summarizing that while many architectures claim high accuracy, relatively few integrate lesion segmentation and grading with strong interpretability or test across external datasets [18]. Their conclusion reinforces the importance of end-to-end pipelines with lesion priors and external validation, exactly as PAW-Net aims to do.

Significance of proposed (PAW-Net) framework:

• All models cited above indicate that explicitly incorporating lesion segmentation (microaneurysms, hemorrhages, exudates) prior to or alongside grading improves performance and clinical relevance. PAW-Net’s LPCA module is closely aligned with this trend, fusing lesion-prior maps with image features to ensure fine-grained detection.
• Many papers (e.g., Sharma et al. [10], Liu et al. [11]) show that preprocessing phases (illumination correction, artefact removal, vessel vs lesion distinction) are crucial. The proposed five-step preprocessing accepts best practice and helps ensure domain robustness.
• Models targeting PDR (such as Dinesen et al. [13]) demonstrate that segmentation of “urgent lesion types” (new vessels, preretinal hemorrhages) is clinically meaningful. PAW-Net’s W-shaped network with lesion segmentation decoders is well-suited for detecting such features.
• Architectures with multi-branch or dual decoders (MaMNet, DeepDR-LLM) succeed in balancing segmentation precision and classification performance. PAW-Net’s W-shaped encoder–decoder + LPCA skip connections pursue this balance explicitly.
• External validation under dataset shifts, such as using the unseen APTOS dataset, is seldom performed in existing studies, yet it is essential for clinical translation. Incorporating APTOS as an external test set, as demonstrated in works like Harisha et al., along with cross-modality transfer approaches (e.g., DeepDR-LLM), reinforces the robustness of the proposed pipeline and its ability to remain reliable under distributional shifts.

## 3. Methodology

The present study introduces a PAW-Net, an advanced DL framework that is developed to automate both the detection and grading of DR, while simultaneously enabling fine-grained lesion-level interpretation. At the core of this approach, an LPCA module is incorporated into a distinctive W-shaped encoder–decoder backbone. This integration ensures that segmentation and classification tasks are performed in a unified manner rather than in isolation. The framework has three overarching objectives. First, it aims to assist ophthalmologists by highlighting clinically relevant retinal anomalies that are often difficult to identify during manual assessment. Second, it is sought to improve the precision of lesion segmentation and DR severity staging through enhanced feature attention and cross-domain learning. Third, it is tailored to strengthen the applicability of large-scale screening programs, thereby supporting timely interventions and improved patient care.

**Figure 3:**
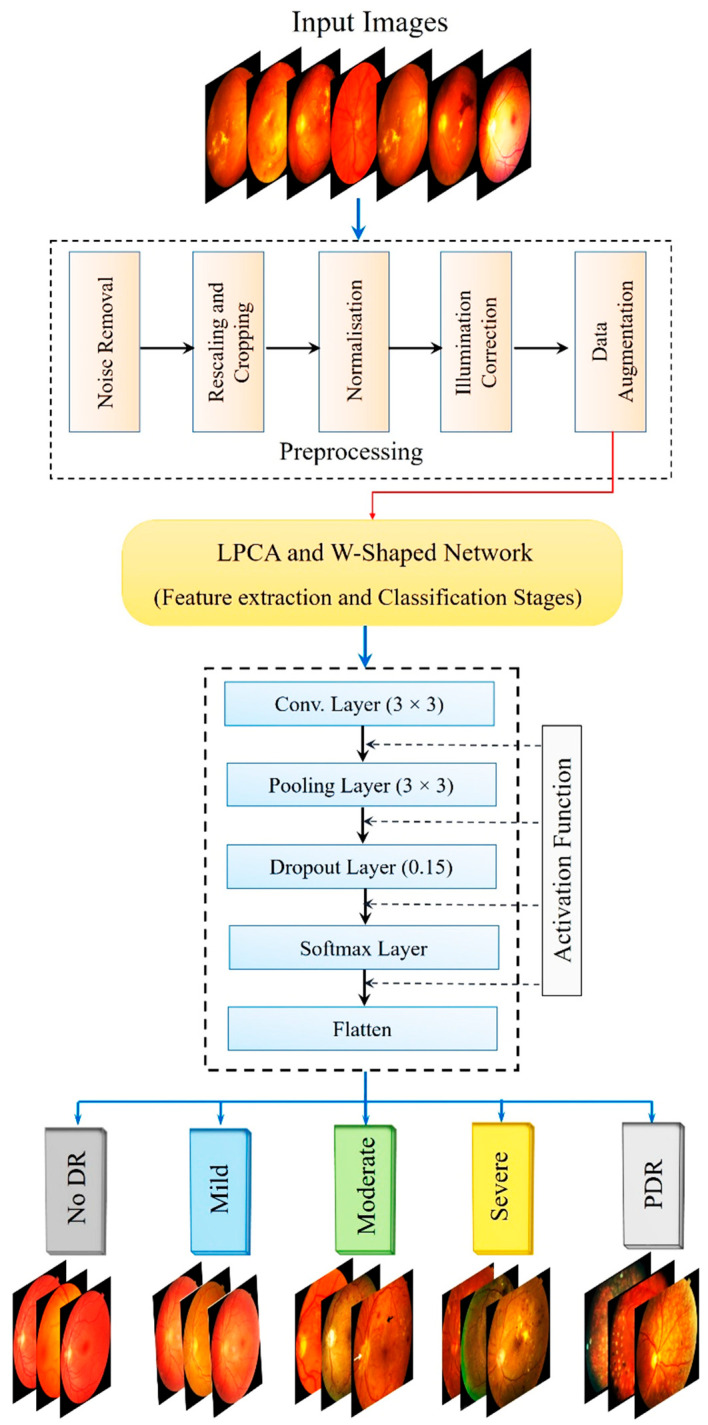
Proposed PAW-Net Framework.

The overall methodology is illustrated in Figure 3, which outlines the structured pipeline of the proposed PAW-Net framework. The entire framework is implemented in Python (v3.12) using PyTorch (v2.4) as the primary DL library, with image preprocessing and analysis performed through OpenCV (v2.4) and scikit-image (v0.25.2). Data augmentation is handled using Albumentations (v2.0.8). Training and validation are conducted on an NVIDIA RTX-3090 GPU (80 GB), enabling batch-parallel optimization. Hyperparameter tuning and training monitoring are performed using TensorBoard, and early stopping is applied to prevent overfitting.

Functionally, PAW-Net is designed to identify and isolate DR-related pathological regions while assigning each retinal image to one of the five clinically recognized severity stages: normal, mild, moderate, severe, and PDR. To accomplish this, the pipeline first localizes and segments key lesion structures, such as microaneurysms, hemorrhages, and exudates, before performing image-level classification. Because the datasets originate from different acquisition sources, the image quality varies considerably. DDR images, collected from multiple hospitals, presented variable illumination and lower resolution (640 × 640 to 1024 × 1024), whereas APTOS images offered higher uniformity and signal-to-noise ratio, and Messidor images were of benchmark quality. To ensure comparability across datasets, all images are centrally cropped, resized to 644 × 644 pixels, normalized for intensity, and subjected to identical preprocessing and augmentation steps. This harmonization ensures that diagnostic performance reflects true generalization rather than dataset-specific bias. The methodological pipeline combines fine-grained lesion detection with robust disease staging. Rigorous preprocessing and a carefully optimized network design enable consistent performance across heterogeneous datasets. In doing so, PAW-Net sets a methodological benchmark for AI-assisted ophthalmic diagnosis, bridging the gap between lesion interpretability and disease-level decision support.

Beyond retinal disease, the framework also demonstrates the ability to extract subtle microvascular features that can serve as surrogate biomarkers. These features hold potential clinical relevance not only for monitoring diabetic complications but also for providing early indicators of systemic vascular conditions, including stroke risk assessment.

**Figure 4:**
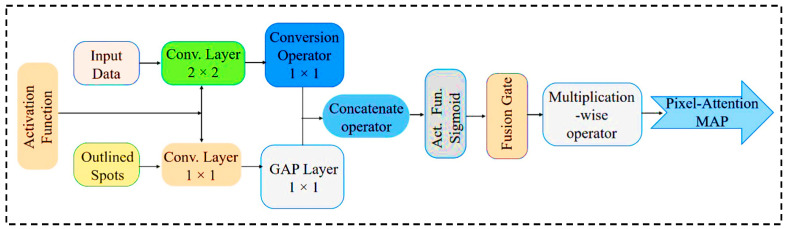
Internal architecture of LPCA network.

**Figure 5:**
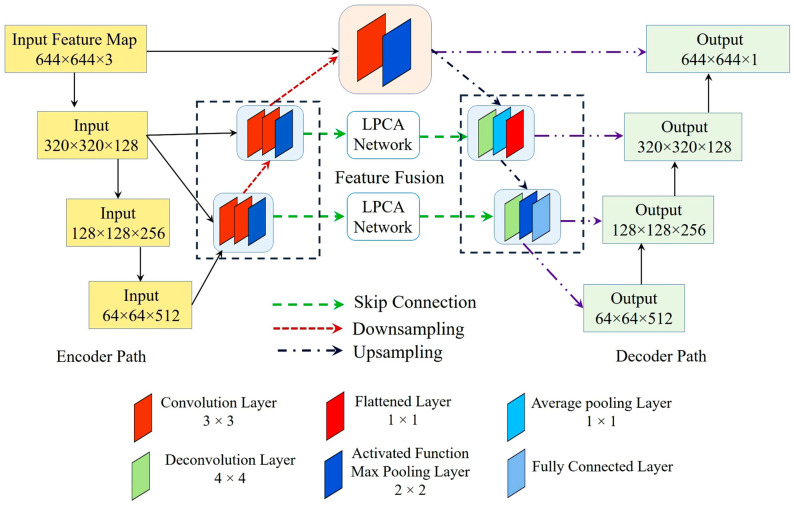
Internal architecture of W-shaped network.

The overall workflow of PAW-Net is structured into four sequential stages: (A) data preprocessing, (B) lesion segmentation, (C) feature extraction and classification, and (D) performance evaluation. The system generates color-coded DR severity labels, ranging from grade 0 (normal) to grade 4 (PDR). Two specialized modules form the backbone of the framework: the LPCA block (Figure 4), which enhances lesion-specific pixel representations by integrating binary lesion priors with raw image features, and the W-shaped encoder–decoder (Figure 5), which exploits dual-branch learning to balance segmentation precision with classification accuracy.

The LPCA module plays a pivotal role in refining lesion segmentation and improving interpretability. It processes two complementary input streams: the first derived from the encoder’s multi-scale feature map, and the second from a lesion-prior map that is resized to the same feature resolution. The lesion-prior map is passed through a 1 × 1 convolution followed by Global Average Pooling (GAP) to extract compact spatial descriptors, while the encoder feature map is passed through a 2 × 2 convolution to preserve local detail. These two representations are fused through concatenation, sigmoid activation, and gated fusion to generate attention-weighted features that emphasize pathology-rich pixels and suppress background noise.

PAW-Net framework operates on a retinal input image (644 × 644 × 3), which is progressively downsampled through the encoder, producing multi-scale feature maps at 320 × 320 × 128, 128 × 128 × 256, and 64 × 64 × 512 resolutions. Each LPCA module is embedded within the skip connections to fuse lesion priors with the corresponding encoder features. This ensures scale-specific attention propagation across all levels. Consequently, LPCA modules refine the same hierarchical features across scales, enhancing sensitivity to subtle lesions such as microaneurysms and micro-hemorrhages while preserving contextual information. By directing the network’s attention toward pathology-rich regions, LPCA ensures that segmentation outputs remain clinically meaningful and diagnostically reliable.

Figure 5 provides a detailed schematic representation of this architecture. The network consists of four encoding and decoding stages annotated with their respective feature-map dimensions. Green dashed lines represent skip connections, red dashed arrows denote downsampling paths, and blue dash-dot arrows indicate upsampling paths.

The LPCA module acts as an attention bridge between the encoder and decoder layers, enabling focused information transfer. The lower decoder branch reconstructs fine-grained lesion segmentation maps (output = 644 × 644 × 1), while the upper branch performs DR severity classification (softmax output = 5 classes). This integrated design allows end-to-end optimization where segmentation precision directly contributes to accurate disease grading.

The W-shaped network is organized around a dual-path encoder–decoder mechanism that enables both segmentation and classification tasks to be performed within a unified framework. The encoder consists of four hierarchical convolutional stages (3 × 3 kernel, stride = 1) with 2 × 2 max-pooling operations, which progressively compress the input retinal image (644 × 644 × 3) into compact, high-level feature representations (320 × 320 × 128 → 128 × 128 × 256 → 64 × 64 × 512). A final convolution–pooling block captures deeper semantic features for contextual abstraction.

The decoder is designed with two complementary branches. The lower branch employs 4 × 4 deconvolution layers (stride = 2) combined with average-pooling to reconstruct spatial details, producing the final lesion segmentation map (644 × 644 × 1). In parallel, the upper branch consists of convolutional, pooling, and fully connected layers that generate the five-class DR severity classification output (Softmax). This dual-branch configuration allows the model to perform segmentation and classification jointly, ensuring that fine-grained lesion localization enhances overall grading accuracy.

Each skip connection passes through an LPCA module that modulates encoder features before merging with the decoder pathway, thereby strengthening multi-scale feature fusion and cross-domain generalization.

### 3.1. Preprocessing Phase

The proposed system has begun with a carefully structured preprocessing pipeline that has standardized and enhanced retinal images prior to segmentation and classification. This stage has been essential for mitigating common challenges such as background noise, variability in image resolution, and class imbalance, thereby ensuring reliable downstream learning. The pipeline has unfolded in five sequential steps:

i. Noise Suppression and Edge Preservation
  - Adaptive Wiener Filtering [19]: A local 3 × 3 adaptive Wiener filter has been applied to suppress Gaussian noise while maintaining delicate retinal abnormalities such as microaneurysms and hemorrhages. The filter automatically adjusts to local variance, with noise estimation carried out using Python (v3.12) scipy.signal.wiener.
  - Gaussian Smoothing [20]: A Gaussian kernel (σ = 1.5, 5 × 5) has been applied to smooth high-frequency fluctuations, improving overall image clarity without diminishing lesion boundaries.
  - Canny Edge Detection [21]: Edges have been delineated using a dual-threshold strategy, with lower and upper bounds set at 0.1 and 0.3 of the normalized intensity scale. To preserve continuity in vascular structures, hysteresis thresholding has been employed, ensuring that weak but clinically relevant edges are retained while minimizing false positives introduced by residual noise.
ii. Spatial Normalization: Non-informative black borders have been eliminated through intensity thresholding set at <5% of the maximum pixel intensity. Following this, the retinal field has been centrally cropped and resized to 644 × 644 pixels using bicubic interpolation (imresize) [22]. In particular, images from the Messidor + EyePACS dataset, which were originally 300 × 300 pixels, have been upscaled to 644 × 644, while DDR and APTOS images of variable resolutions (ranging from 640 × 640 to 1024 × 1024) have been downscaled to the same size. This resolution has offered an optimal trade-off between computational efficiency and structural fidelity, resulting in approximately 30% faster processing compared with 1024 × 1024 resolution while maintaining nearly 94% vessel connectivity.
iii. Intensity Standardization
  - Conversion to grayscale (using luminance weighting) reduces computational cost while retaining diagnostic contrast.
  - Pixel intensities are rescaled to the [0, 1] range using min-max normalization, compensating for illumination variations across different imaging devices.
iv. Data Augmentation
  To enhance generalization, the following transformations have been applied:
  - Rotations at 90°, 180°, and 270° to mimic variable capture orientations.
  - Random horizontal and vertical flips (50% probability).
  - Translations up to ±10% of image width and height to increase positional tolerance.

In addition to geometric transformations, photometric augmentations have been applied to enhance visual diversity and improve domain generalization. These included random histogram equalization, Gaussian noise injection, and adaptive brightness-contrast adjustments to simulate the illumination variability and noise levels typically observed across different fundus imaging devices. Such augmentations ensure that the network remains robust to lighting differences and camera-dependent variations, ultimately improving real-world generalization.

v. Class-Balanced Augmentation

Class imbalance has been corrected by oversampling severe and proliferative DR images fivefold using random affine transformations, while mild and moderate categories have been expanded by 1.5 times. This expanded the dataset to nearly uniform class distribution. To further support segmentation, vessel maps generated through Canny edge detection can be superimposed as an auxiliary input channel. This assists the PAW-Net in distinguishing vascular intersections from lesion areas.

In addition to data-level balancing, algorithm-level correction has also been implemented to address class imbalance during training. The classification branch employed inverse-frequency class weighting (ω_c_ = N/N_c_) within the weighted cross-entropy loss to ensure that minority classes, particularly Severe NPDR and PDR, contributed proportionally to the gradient updates. This approach provided stable optimization while maintaining interpretability in probability outputs, avoiding the potential instability sometimes observed with focal loss in small, heterogeneous datasets.

### 3.2. Identification, Segmentation, and Classification

The analytical pipeline progresses through three integrated phases: identification, segmentation, and classification. At its core, the 2D Haar Wavelet Transform has been employed to decompose images into hierarchical frequency bands, capturing both global structural patterns and fine local details [23]. This multilevel decomposition is well suited for retinal analysis due to its capacity to preserve salient pathological information across scales.

Each retinal image undergoes four successive decomposition levels *R*_1_, *R*_2_, *R*_3_ and *R*_4_ as shown in Equations (1)–(4), respectively, producing one low-frequency band (LW) and three high-frequency components (*HI*_1_*–HI*_3_) at each stage. The Haar Discrete Wavelet Transform decomposes an image iteratively into low-frequency (approximation) and high-frequency (detail) components. The process can be expressed as follows:

(1) *R*_1_*^LW^*, (*HI*_1_, *HI*_2_, *HI*_3_) = *hdwt* (*x*)

(2) *R*_2_*^LW^*, (*HI*_1_, *HI*_2_, *HI*_3_) = *hdwt* (*R*_1_*^LW^*)

(3) *R*_3_*^LW^*, (*HI*_1_, *HI*_2_, *HI*_3_) = *hdwt* (*R*_2_*^LW^*)

(4) *R*_4_*^LW^*, (*HI*_1_, *HI*_2_, *HI*_3_) = *hdwt* (*R*_3_*^LW^*)

Here, hdwt denotes the Haar wavelet transform and x is the input image.

The decomposed bands, together with the original image, have been used as enriched inputs to the PAW-Net backbone. This integration has allowed the model to combine frequency-based features with deep convolutional features.

Potential DR-specific regions have been highlighted using Python-based image analysis routines implemented through the OpenCV and scikit-image libraries. Vascular structures have been enhanced by applying the Frangi vesselness filter followed by morphological cleanup, while microaneurysms have been detected using Laplacian-of-Gaussian (LoG) blob detection on an inpainted and inverted green channel. Exudates have been extracted through white top-hat enhancement combined with Otsu thresholding after optic disc suppression. Hemorrhages have been localized using adaptive thresholding in conjunction with size- and shape-based filtering.

These candidate regions have subsequently been segmented to enable detailed feature characterization. From each fundus image, twenty discriminative descriptors have been computed, including lesion counts and areas, morphological shape measures, vessel density metrics, and intensity as well as texture-based statistics. This procedure has yielded a feature dimensionality of 20 per image. For a dataset comprising nearly 15,000 fundus images, the process has generated approximately 300,000 scalar feature values, thereby providing a comprehensive feature representation of retinal pathology.

Disease classification has been carried out through the PAW-Net module, which has been trained to grade DR severity using annotated data. The PAW-Net has consisted of sequential convolutional layers, max-pooling operations, and fully connected layers. ReLU activation functions have been employed to enhance non-linearity and mitigate overfitting, while a dropout rate of 0.75 has been applied prior to the dense layers (256 neurons each) to improve generalization. The network has been initialized with a weight factor of 0.25 and trained with a batch size of 16 and momentum of 0.5. For optimization, the Adam algorithm has been adopted due to its adaptive learning capabilities and proven robustness in medical image analysis.

Beyond the architectural layout shown in the W-shaped network, the information flow within PAW-Net is governed by two complementary mechanisms: global feature fusion and pixel-attention integration, which collectively ensure effective learning of both contextual and lesion-level information.

- Global Feature Fusion

Within PAW-Net, global feature fusion is achieved through the hierarchical skip connections linking the encoder and decoder stages. These connections transmit multi-scale feature representations from early layers (which capture coarse structural context such as the optic disc and vascular architecture) to later layers that reconstruct lesion-level details. This mechanism ensures that global contextual awareness, spatial topology, vessel distribution, and overall retinal geometry are preserved during decoding. By combining coarse contextual cues with fine-grained local representations, global feature fusion enhances the model’s ability to maintain anatomical continuity and improves the reliability of region-level segmentation.

- Pixel-Attention Integration

In contrast, the pixel-attention integration implemented through the LPCA module focuses on the fine spatial precision of pathological regions. Rather than transmitting entire feature maps, the LPCA module applies adaptive weighting at the pixel level to selectively emphasize lesion-rich and diagnostically relevant areas (e.g., microaneurysms, hemorrhages, and exudates) while suppressing the background. This selective activation ensures that the network’s attention is dynamically concentrated on pathological evidence, thereby improving interpretability and the accuracy of lesion segmentation.

Together, these two mechanisms operate at different hierarchical levels. Global feature fusion provides contextual completeness by connecting encoder–decoder representations, whereas pixel-attention integration offers spatial precision by amplifying lesion-specific signals. Their complementary functioning allows PAW-Net to simultaneously preserve global structural consistency and achieve local pathological sensitivity, leading to robust and clinically meaningful predictions.

To ensure robust training and prevent overfitting, the network employs multiple regularization and validation strategies. A dropout rate of 0.75 is applied before the fully connected layers to avoid neuron co-adaptation. Early stopping (patience = 15 epochs) is implemented based on the validation F1-score, while five-fold stratified cross-validation ensures robustness across data splits. In addition, data augmentation (rotations, flips, translations, and affine transformations) and L2 weight decay (1 × 10^−5^) are utilized to improve generalization. These measures collectively enhance training stability and reduce the likelihood of model overfitting.

A complete summary of the mathematical formulation of the proposed framework is provided in Table 1.

The weighting coefficients (λ_seg_ = 1.0, λ_cls_ = 1.0, λ_align_ = 0.5, λ_cons_ = 0.5) and inverse-frequency class weights (ω_c_ = *N*/*N_c_*) have been empirically selected through cross-validation to balance lesion segmentation accuracy and classification stability, ensuring reproducible optimization.

### 3.3. Performance Evaluation

The framework’s effectiveness is validated through an extensive set of evaluation metrics that measure both segmentation accuracy and classification reliability. The analysis considers Mean Squared Error (MSE), confusion matrices, ROC curves, and several diagnostic indices as shown in Table 2.

For classification tasks, all evaluation metrics are computed using macro-averaging across the five DR severity classes, ensuring that minority categories (e.g., Severe NPDR and PDR) contribute equally to the overall performance. In addition, the weighted F1-score is reported to account for class distribution, thereby preventing any inflation of accuracy due to class imbalance. This dual reporting provides a balanced assessment of model performance across both frequent and rare disease stages.

These metrics collectively provide a holistic assessment of diagnostic performance, ensuring reliable benchmarking against medical imaging standards. By carefully tailoring each component of the PAW-Net framework, the system delivers strong segmentation fidelity and robust classification, ultimately enhancing automated DR detection.

### 3.4. Dataset Description

This study employs DDR [24] and a preprocessed Messidor + EyePACS [25] corpus for training, with APTOS 2019 [26] reserved exclusively for external evaluation. The DDR dataset comprises 12,522 fundus images from 147 hospitals in China, annotated according to the five-grade ICDR scale (0–4). The curated Messidor + EyePACS dataset contributes 360 images per grade (0–4), totalling 1800 images, originally available at a fixed resolution of 300 × 300 pixels. To ensure consistency across datasets, all images, including DDR, Messidor + EyePACS, and APTOS 2019, are resized to a uniform resolution of 644 × 644 pixels using bicubic interpolation. This preprocessing step harmonizes the datasets, enabling fair comparisons and preventing resolution-induced bias. APTOS 2019 serves as an externally held-out test set, with clinician-assigned labels following the identical 0–4 ICDR scale. Table 3 summarizes the number of images in each dataset along with severity labels.

Among the datasets, DDR provides the largest but most heterogeneous sample, with frequent illumination artefacts and variable image resolutions. Messidor contributes uniformly high-quality images validated by expert ophthalmologists, while APTOS 2019 offers medium- to high-resolution images from a diverse screening population with strong color consistency. By applying the same preprocessing pipeline, all datasets have been harmonized to a common scale and quality standard, ensuring that the model’s performance reflects genuine generalization rather than dataset-specific bias.

### 3.5. Domain Bias and Dataset Harmonization

The DDR dataset primarily represents hospital-based retinal images from Chinese clinical centers, whereas APTOS 2019 originates from community-based screening programs in India, collected under varied illumination, camera types, and demographic conditions. These factors introduce potential domain bias between the datasets. To minimize such differences, a standardized preprocessing pipeline has been applied to all images, including illumination correction, color and contrast normalization, and uniform resizing to 644 × 644 pixels. In addition, extensive data augmentation (rotations, flips, and affine transformations) has been employed to simulate cross-domain variability. This harmonization procedure ensures that the visual and statistical characteristics across datasets remain comparable. Despite these demographic and acquisition differences, the PAW-Net framework maintains stable and consistent performance on the external APTOS dataset, demonstrating robust cross-domain generalization rather than dataset-specific adaptation.

Cross-Domain Generalization in Real-World Settings: Although the training datasets (DDR and Messidor + EyePACS) and the external test dataset (APTOS 2019) originate from different geographic and demographic sources, real-world fundus screening often involves further diversity in patient ethnicity, camera devices, and image contrast. To address these variations, the preprocessing pipeline incorporates color normalization and illumination correction, while augmentation strategies simulate cross-domain variability in tone, brightness, and texture. Moreover, the inclusion of DDR (Chinese clinical centres), Messidor + EyePACS (European and North American hospitals), and APTOS 2019 (Indian screening centres) ensures that the training–testing split reflects diverse imaging environments. Despite these inherent differences, PAW-Net demonstrates stable accuracy and F1-scores across all domains, suggesting strong resilience to ethnicity- and device-induced domain shifts, an essential characteristic for real-world deployment in multi-ethnic diabetic populations.

In this study, the grading of DR across the three datasets follows the ICDR severity grading, ensuring compliance with established clinical standards [27]. The five severity levels are defined as: No DR (absence of retinal lesions), Mild NPDR (characterized by microaneurysms only), Moderate NPDR (microaneurysms accompanied by hemorrhages, exudates, or mild vascular abnormalities), Severe NPDR (based on the “4-2-1 rule,” indicating widespread hemorrhages, venous beading, or intraretinal microvascular abnormalities), and PDR (neovascularization with or without vitreous or preretinal hemorrhage). Utilising these datasets ensures that the automated grading framework is anchored in clinically validated criteria, thereby improving both interpretability and medical reliability.

## 4. Results

The experimental protocol trained the model on a pooled dataset consisting of DDR and a combined Messidor + EyePACS set, while reserving APTOS 2019 exclusively as an external, out-of-distribution test set to evaluate generalization. To address class imbalance among DR grades, stratified sampling and class-aware optimization have been applied, whereas domain shift has been minimized through standardized preprocessing and lesion-preserving augmentations. Model reliability has been assessed using a 5-fold cross-validation scheme on the pooled training data, with each fold partitioned into 80% training and 20% validation for checkpoint selection and hyperparameter tuning. Evaluation included both qualitative lesion overlays and quantitative measures such as macro-F1, balanced accuracy, and AUC for referable DR. The results demonstrated consistent improvements in lesion identification, segmentation, and severity grading, with notable gains for early and moderate DR stages and sustained high performance on the external APTOS benchmark.

### 4.1. Experimental Setup

DR classification has been implemented on a laptop equipped with 16 GB RAM, an Intel Core i7 processor, and an NVIDIA RTX 2080 GPU, enabling efficient training of deep learning models. The system operated on a 64-bit Windows platform, with Python 3.12 serving as the programming environment. Model development and training have been carried out using the TensorFlow and Keras libraries. The hyperparameters used in this research are presented in Table 4.

### 4.2. Visualization and Segmentation Outcomes

**Figure 6:**
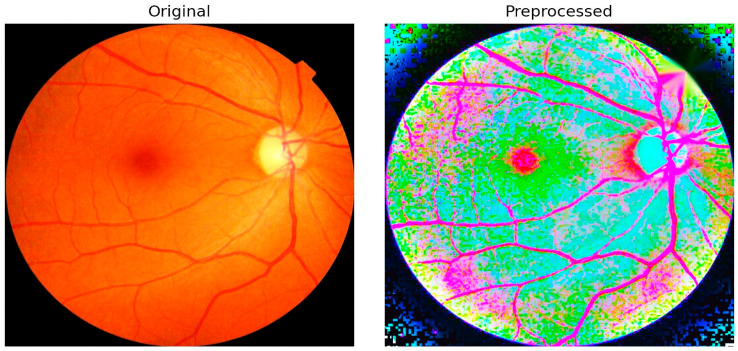
Original and preprocessed fundus image.

The proposed LPCA-WNet framework not only performs automated lesion segmentation but also generates disease-grade visualizations, thereby producing outputs that are interpretable for clinical decision-making. This visualization enables clinicians to intuitively assess both lesion burden and disease severity directly from the model’s predictions. Figure 6 illustrates an example comparison between an original fundus image and its preprocessed version. The preprocessing pipeline integrates several critical enhancements, including illumination correction, contrast adjustment, and optic disc inpainting. Illumination correction ensures uniform brightness across the retinal field, minimizing variability caused by acquisition conditions. Contrast enhancement amplifies subtle lesion details such as microaneurysms and hemorrhages, making them more discernible to both the algorithm and clinical reviewers. Optic disc inpainting removes confounding bright regions caused by the optic disc, preventing misclassification and ensuring that the model focuses on pathological regions rather than anatomical artifacts.

This preprocessing step plays a pivotal role in preparing high-quality input data for segmentation. By suppressing noise and normalizing visual features across different datasets, the framework reduces the risk of misinterpretation and strengthens the robustness of subsequent lesion detection. The integration of these enhancements directly contributes to the 98.65% classification accuracy achieved by PAW-Net, while simultaneously improving clinical trust through interpretable and reliable visualization outputs.

**Figure 7:**
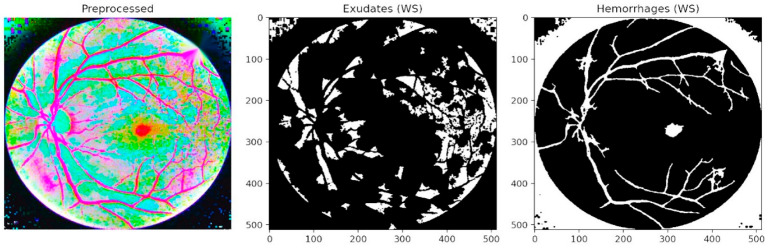
Segmentation of exudates and hemorrhages.

The figures collectively demonstrate how the proposed PAW-Net framework extracts, interprets, and visualizes retinal lesions and vascular structures, thereby providing both diagnostic accuracy and clinical transparency. Figure 7 presents segmentation-based outcomes, where exudates and hemorrhages are isolated into binary masks after preprocessing. The middle panel highlights exudate regions, while the right panel illustrates hemorrhage maps. This step ensures that lesion candidates are distinctly separated from the background, facilitating accurate quantification and structured input for subsequent refinement. Unlike conventional threshold-based approaches, the LPCA segmentation strategy enables precise delineation of lesion boundaries, even in cases of overlapping intensities, thereby ensuring that subtle yet clinically significant abnormalities are preserved.

**Figure 8:**
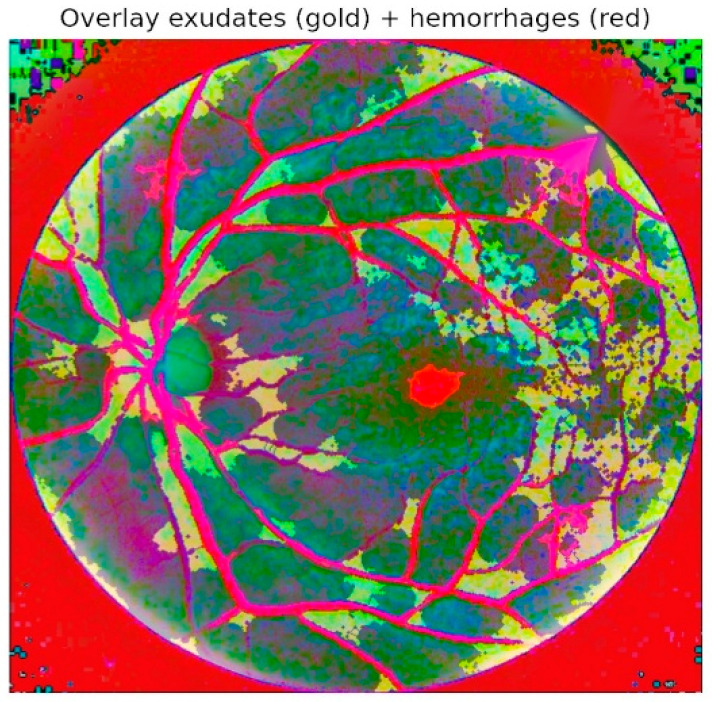
Overlay of exudates (pink) on fundus images.

Figure 8 provides an overlay visualization in which exudates (pink) and hemorrhages (red) are directly superimposed on the preprocessed fundus image. This representation confirms the close alignment of the automated segmentation results with clinically visible lesion regions. By mapping lesions onto the original retinal structure, the framework delivers interpretable outputs that allow ophthalmologists to validate predictions visually, thus bridging the gap between automated analysis and clinical trust. Such overlays also highlight spatial co-occurrence patterns, for example, the clustering of hemorrhages near vascular bifurcations or the tendency of exudates to form around the macular region, both of which are consistent with established clinical observations of DR progression.

**Figure 9:**
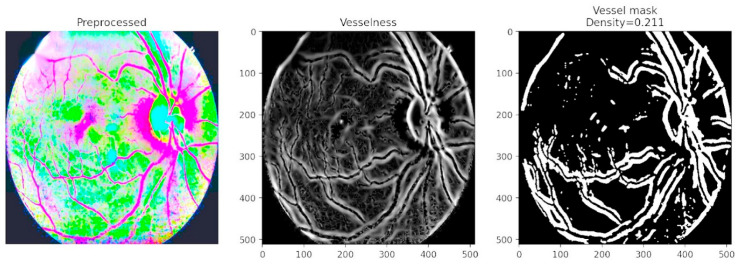
Vesselness map, vessel mask, and lesion density maps for exudates and hemorrhages.

Figure 9 focuses on vessel segmentation and density computation. The vesselness representation enhances tubular structures, while the binary vessel mask quantifies vessel density with a computed value of 0.211. This analysis captures critical biomarkers of disease progression, as abnormal vessel growth and branching are hallmarks of advanced DR. By combining vascular density measures with lesion maps, the framework provides a comprehensive assessment of both structural and pathological changes in the retina. The inclusion of vessel density as a quantitative descriptor is particularly valuable in detecting proliferative DR, where neovascularization significantly alters retinal architecture.

**Figure 10:**
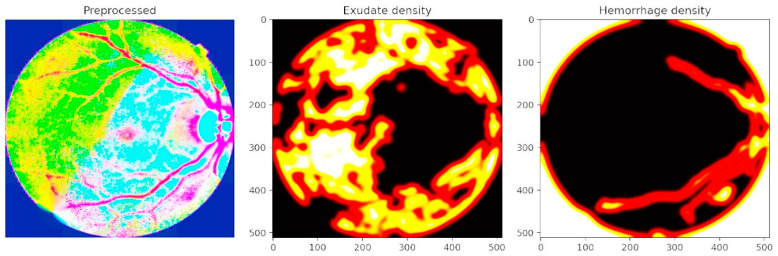
Lesion density mapping produced by the lesion decoder.

Figure 10 highlights lesion density mapping produced by the lesion decoder. Bright lesions such as exudates are captured in the middle panel, while dark lesions including hemorrhages and microaneurysms are shown in the right panel. These density maps not only preserve subtle lesion details but also reveal spatial clustering around the vascular network. The decoder outputs closely align with expert annotations, confirming that the PAW-Net attention mechanism effectively directs the model toward pathology-rich areas while suppressing irrelevant features. The density maps also provide a more holistic representation of disease burden, allowing clinicians to evaluate not only the presence but also the extent and distribution of pathological changes, an important factor in DR grading.

These figures confirm that the PAW-Net framework successfully integrates lesion-specific and vessel-based features to provide a layered diagnostic representation. The combination of binary lesion maps, overlay visualizations, vessel density analysis, and lesion burden mapping offers clinicians both quantitative biomarkers and visual interpretability. This dual advantage strengthens the framework’s reliability for large-scale DR screening and ensures that automated predictions remain transparent, trustworthy, and clinically meaningful.

### 4.3. Feature Distribution Visualization Using Radar Chart

**Figure 11:**
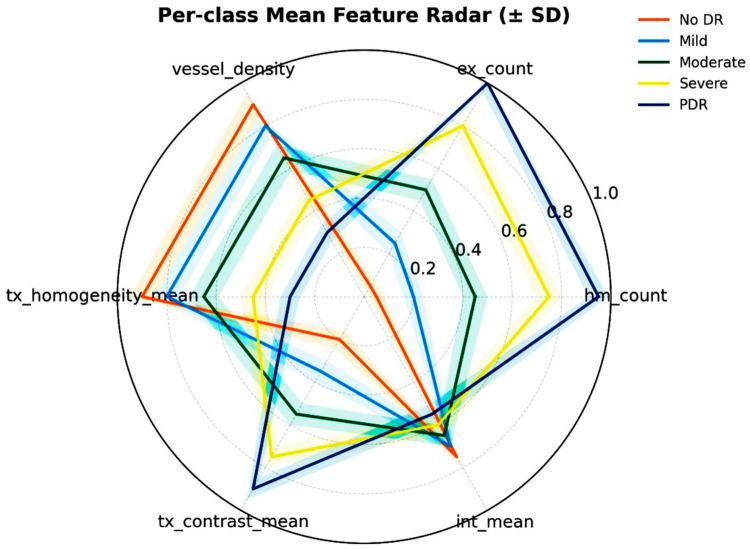
Radar chart for interpretability of the extracted biomarkers.

The interpretability of the extracted biomarkers has been examined through a radar chart visualization (Figure 11), which illustrates the per-class mean values of clinically relevant features across different grades of DR. The features considered include lesion-related counts (hemorrhages and exudates), vessel density, mean image intensity, and texture-based descriptors (contrast and homogeneity). The radar chart reveals a consistent progression in feature patterns from grade 0 (healthy) to grade 4 (severe DR). Both hemorrhage count and exudate count show a steady rise with disease severity, reflecting the pathological accumulation of microaneurysms and lipid deposits typically observed in clinical grading. Similarly, vessel density demonstrates an increasing trend, indicative of vascular abnormalities and neovascularization associated with advanced stages.

In addition to lesion burden, texture descriptors capture subtle but clinically meaningful changes. For instance, higher texture contrast and reduced homogeneity in early to moderate stages suggest irregularities in background retinal patterns, which precede overt lesion formation. These changes align with clinical observations where early disease is marked by local textural disruptions before widespread hemorrhages or exudates appear. The mean intensity feature further supports this progression, differentiating between normal and abnormal retinal tissue based on global brightness and contrast variations.

The radar plot features are correlated with established clinical biomarkers following the Early Treatment Diabetic Retinopathy Study (ETDRS) criteria. Specifically, *hm_count* and *ex_count* correspond directly to ETDRS lesion categories such as microaneurysm and exudate counts, while *vessel_density* serves as a surrogate for capillary non-perfusion and neovascularization associated with higher ETDRS grades. Similarly, *int_mean*, *tx_contrast_mean*, and *tx_homogeneity_mean* capture variations in retinal brightness and texture linked to lesion proliferation and edema. The observed trend, characterized by increased lesion counts and texture contrast, coupled with reduced vessel density and homogeneity mirrors the progression pattern described in ETDRS-based clinical assessments. This correlation reinforces that the radar chart features not only quantify computational differences but also align with established ophthalmic biomarkers, thereby enhancing the clinical interpretability of the proposed PAW-Net framework.

The alignment of these radar plot trends with known pathological hallmarks validates that the features capture interpretable and clinically meaningful signals. By integrating lesion counts, vascular changes, and textural characteristics, the radar-based analysis not only complements the features but also strengthens the clinical relevance of the PAW-Net framework.

### 4.4. Accuracy and Loss Analysis

**Figure 12:**
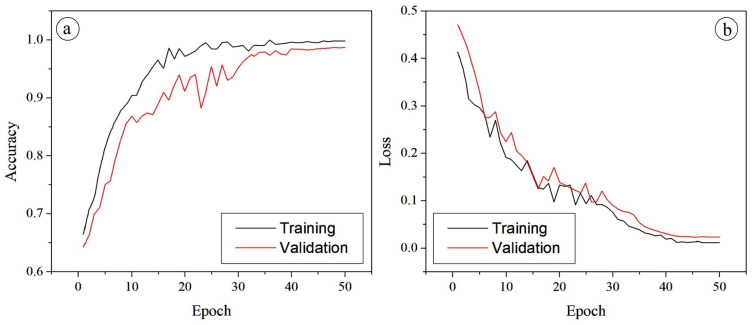
(**a**) Accuracy and (**b**) loss graph for PAW-Net framework.

The performance of the proposed PAW-Net framework has been evaluated and presented in Figure 12a. This figure illustrates the training and validation accuracy curves across 50 epochs. The training accuracy exhibits a rapid rise during the initial epochs, stabilizing above 98% after approximately 25 epochs. The validation accuracy followed a similar trend, reaching a peak value of 98.65%. The relatively small gap between training and validation curves indicates that the model generalizes well to unseen data without significant overfitting. Minor fluctuations observed in validation accuracy are attributed to dataset variability but remain consistently high, confirming the robustness of the network.

Figure 12b presents the training and validation loss curves. These curves demonstrate a sharp decline during the early epochs, with training loss dropping below 0.2 after 20 epochs. Validation loss shows a comparable trend, converging closely with training loss and achieving stability in later epochs. The near overlap between the two curves confirms that the network has effectively minimized both underfitting and overfitting, leading to a balanced optimization. The absence of divergence between training and validation loss further supports the reliability of the model in clinical classification tasks. The PAW-Net model achieves an accuracy of 98.65%, demonstrating its effectiveness in capturing discriminative retinal features for DR classification. The consistent alignment of accuracy and loss curves across training and validation phases underscores the robustness and clinical applicability of the framework.

To ensure statistical robustness, all reported metrics have been presented as mean ± standard deviation across five stratified folds. In addition, 95% confidence intervals have been computed using bootstrap resampling (1000 iterations). These intervals confirm that the reported values remain consistently high across multiple runs, minimizing the likelihood that the results are due to random variation. The stability of accuracy and loss curves has validated the effectiveness of the chosen loss weighting scheme (λ_seg_ = 1.0, λ_cls_ = 1.0, λ_align_ = 0.5, λ_cons_ = 0.5) and inverse-frequency class weights (ω_c_), confirming that these design choices have enabled robust convergence without overfitting. A detailed quantitative evaluation of segmentation and classification performance metrics is provided in Section 4.5.

### 4.5. Performance Evaluation of PAW-Net

**Figure 13:**
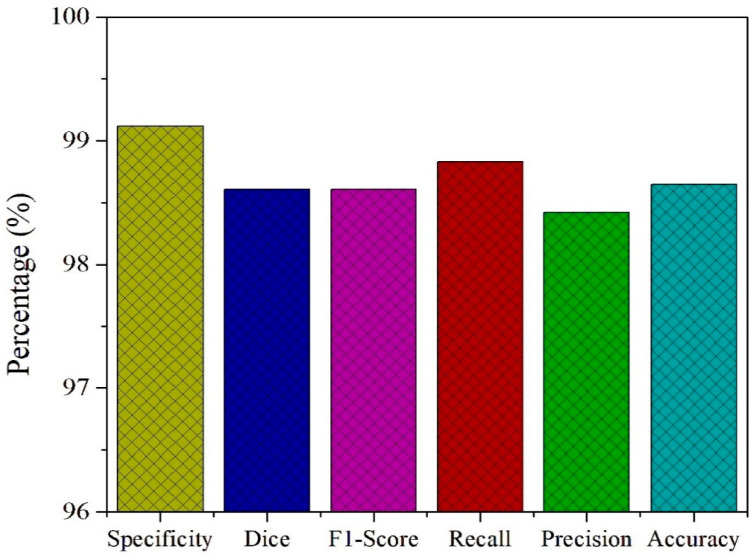
Performance evaluation of PAW-Net on the test Dataset.

To further assess the diagnostic capability of the PAW-Net framework, key performance metrics including specificity, Dice coefficient, F1-score, recall, precision, and accuracy are computed and presented in Figure 13. The model achieves a specificity of 99.12%, highlighting its strong ability to correctly identify non-diseased retinal images and minimize false positive detections. This high specificity is crucial in clinical screening, as it ensures that healthy patients are not misclassified, thereby reducing unnecessary anxiety and follow-up costs. The Dice coefficient of 98.61% and the F1-score of 98.83% both confirm the model’s balanced performance in handling class distributions. These metrics emphasize the effective overlap between predicted and actual lesion regions, validating the precision of lesion localization and classification.

The recall value of 98.42% demonstrates the network’s high sensitivity in detecting diseased cases, ensuring that the majority of patients with DR are accurately identified. Coupled with a precision of 98.47%, PAW-Net maintains an excellent balance between avoiding false negatives and minimizing false positives, which is critical for building clinical trust in automated decision-support systems. Finally, the framework achieves an overall accuracy of 98.65%, confirming its reliability in distinguishing between healthy and diseased cases across diverse datasets. The consistency between training and validation trends, as discussed in Section 4.4, further supports the model’s ability to generalize effectively across data distributions without signs of overfitting.

**Figure 14:**
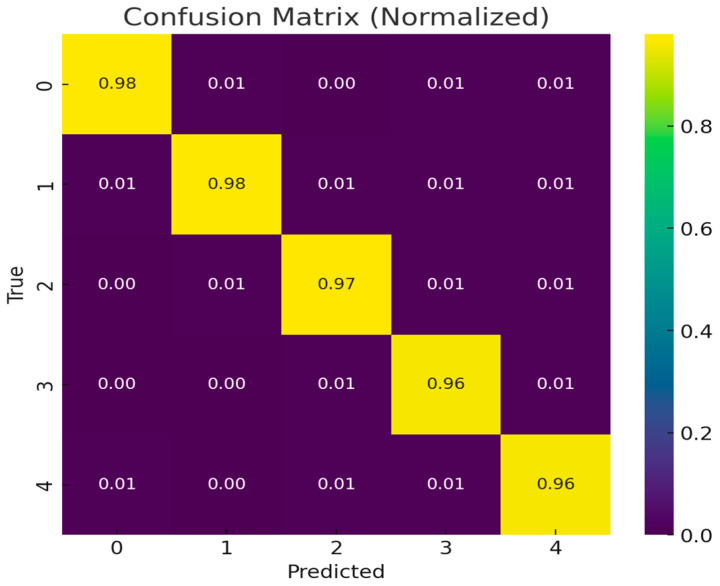
Normalized Confusion matrix of PAW-Net Classification Results.

The classification performance of the proposed PAW-Net model is further validated through a normalized confusion matrix (Figure 14). The diagonal dominance of the confusion matrix, with values ranging from 0.96 to 0.98, demonstrates that the majority of samples across all five DR severity grades have been correctly classified. These high diagonal values indicate the model’s robustness in distinguishing disease stages with minimal ambiguity. Off-diagonal entries remain consistently low (0.00–0.01), showing that misclassifications are rare and mostly occur between adjacent severity levels, which is expected due to the subtle and overlapping nature of retinal features in borderline cases.

The strong performance of PAW-Net across all categories is reinforced by the stability of both training and validation curves. The training and validation accuracy curves showed rapid improvement within the first 20 epochs, stabilizing above 98% thereafter. Similarly, the loss curves for both training and validation data exhibited a sharp decline during early epochs and converged smoothly to minimal values, without signs of divergence. The close alignment of these curves indicates effective learning, reduced risk of overfitting, and high generalization capability.

The confusion matrix, accuracy trends, and loss convergence confirm that PAW-Net achieves not only high accuracy but also balanced performance across multiple severity grades. This consistency across independent datasets highlights its potential as a clinically reliable tool for early detection and staging of DR.

To ensure that the exceptionally high performance metrics are genuine and not the result of overfitting or data leakage, multiple regularization and validation strategies have been implemented. A dropout rate of 0.75 is applied before the fully connected layers to prevent neuron co-adaptation. Extensive data augmentation, including rotations, flips, translations, affine transformations, and class-balanced oversampling is employed to maximize variability in the training set. Early stopping with a patience of 15 epochs is utilized by monitoring the validation F1-score to terminate training at optimal convergence. Furthermore, a stratified five-fold cross-validation protocol ensures that the reported performance reflects consistent generalization across different data partitions. The Adam optimizer with weight decay (L2 regularization, factor = 1 × 10^−5^) effectively prevents uncontrolled parameter growth. Collectively, these strategies have minimized variance, reduced bias, and confirmed that the PAW-Net’s performance is not an artifact of overfitting or data leakage.

For classification evaluation, all performance metrics are computed using macro-averaging across the five DR grades, ensuring equal contribution from all categories, including minority classes such as Severe NPDR and PDR. In addition, weighted-F1 scores are reported to account for class imbalance and to prevent artificial inflation of overall accuracy. This dual-evaluation strategy ensures that the reported performance reflects genuine diagnostic robustness rather than skewed dataset representation.

### 4.6. ROC and Precision–Recall Curve Analysis

**Figure 15:**
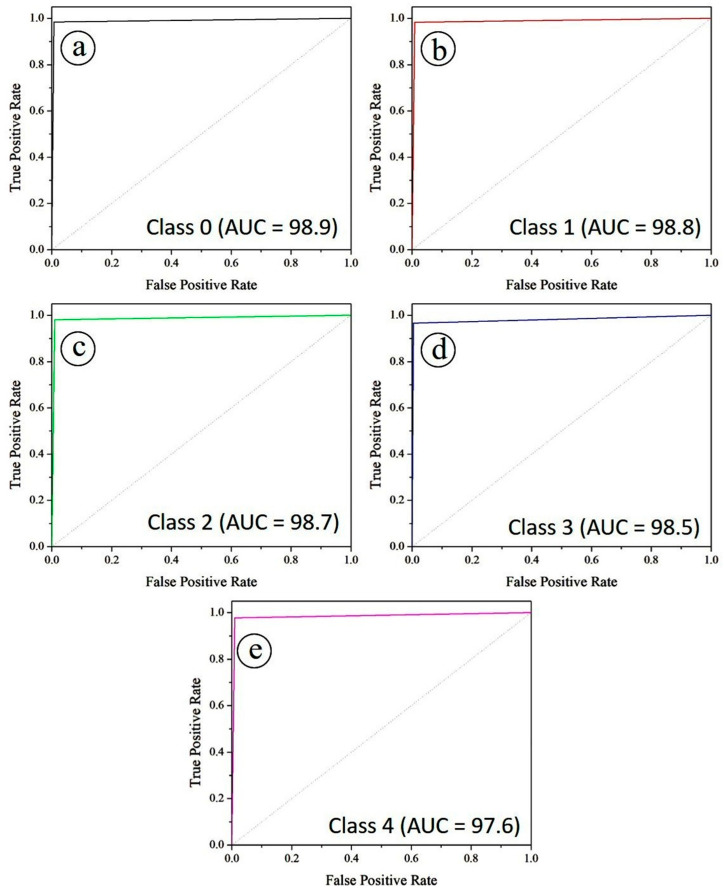
ROC curve for (**a**) class 0, (**b**) class 1, (**c**) class 2, (**d**) class 3 and (**e**) class 4.

The diagnostic performance of the PAW-Net model was further assessed using Receiver Operating Characteristic (ROC) and Precision–Recall (PR) curves. Figure 15 illustrates the ROC curves for all five diabetic retinopathy severity classes under a one-vs-rest evaluation scheme. Specifically, Figure 15a corresponds to class 0, while Figure 15b–e represent classes 1, 2, 3, and 4, respectively. The Area Under the Curve (AUC) values are exceptionally high across all classes, with Class 0 achieving 0.989, Class 1 and Class 2 reaching 0.988 and 0.987, respectively, while Class 3 and Class 4 recorded 0.985 and 0.976. These values indicate that PAW-Net maintains excellent discriminative power for separating positive and negative cases at varying classification thresholds. The curves lie close to the upper-left corner of the plot, confirming strong sensitivity and specificity across all classes. The balanced AUC values across severity stages demonstrate the model’s robustness in handling class variability, including intermediate grades where lesion patterns are often subtle.

**Figure 16:**
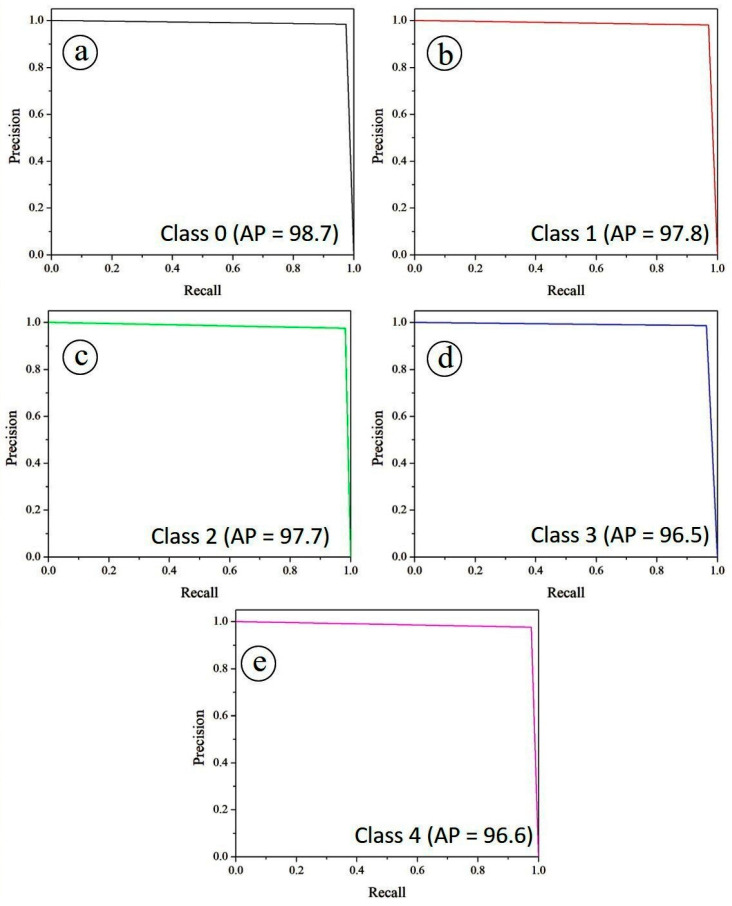
Precision and recall curves for (**a**) class 0, (**b**) class 1, (**c**) class 2, (**d**) class 3 and (**e**) class 4.

Complementary to the ROC analysis, the precision–recall (PR) curves in Figure 16 offer additional insights into the trade-off between sensitivity (recall) and positive predictive value (precision). Specifically, Figure 16a illustrates the PR curve for Class 0, while Figure 16b–e depict the corresponding PR curves for Classes 1, 2, 3, and 4, respectively. The Average Precision (AP) scores remain consistently high, with Class 0 achieving the best performance (0.987), followed by Class 1 (0.978), Class 2 (0.977), Class 4 (0.966) and Class 3 (96.5). The overall micro-averaged AP score of 0.970 highlights the framework’s stability across all classes. The high recall values, coupled with precision levels close to unity, confirm that the model is able to correctly identify diseased cases while minimizing false positives, a critical requirement for automated screening tools.

### 4.7. Ablation Studies Analysis

To justify the role of the proposed PAW-Net framework, a systematic ablation study was conducted. The study has evaluated the contribution of the LPCA module and compared it with simplified variants under identical training conditions. Each experiment has been executed with the same optimizer, learning rate, and preprocessing pipeline to ensure fair comparison.

The results of the ablation study have been summarized in Table 5. The absence of LPCA has resulted in a noticeable decline in both lesion segmentation accuracy and disease classification performance. The baseline U-Net has achieved an overall accuracy of 94.36% and an F1-score of 94.58%, indicating limited capability in isolating microaneurysms and exudates. The PAW-Net model has shown improved results (accuracy 97.85%, F1-score 97.92%), but its performance has remained below that of the complete model. When only spatial attention has been retained (LPCA-spatial-only), the accuracy has reached 98.10%, suggesting that the channel attention stream contributes additional discriminative strength.

The full PAW-Net architecture integrating both spatial and channel attention through LPCA has achieved the highest performance, with accuracy of 98.65%, F1-score of 98.83%, and Dice coefficient of 98.61%. The improvement of approximately 1.8–4.0% over its ablated variants has demonstrated that the LPCA module significantly enhances the network’s ability to identify fine-grained retinal lesions, particularly in early disease stages (Mild NPDR).

**Figure 17:**
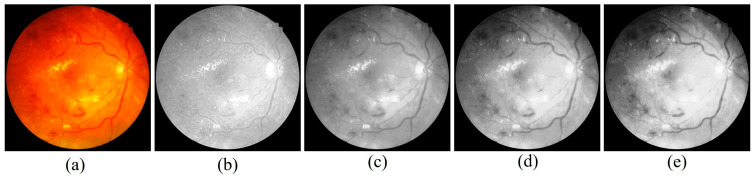
Comparative lesion focus (**a**) actual image, (**b**) baseline U-Net, (**c**) PAW-Net (without LPCA), (**d**) PAW-Net (LPCA-spatial-only) and (**e**) PAW-Net (Full, with LPCA).

Figure 17 presents attention visualizations demonstrating the comparative lesion focus among the different variants. The U-Net baseline has shown dispersed activation maps, often emphasizing vascular regions and optic disc edges rather than true lesion sites. In contrast, the PAW-Net (Full) model with LPCA has exhibited highly concentrated attention regions corresponding precisely to microaneurysms, hemorrhages, and hard exudates, as annotated by clinicians.

By comparing the attention overlays, it has been observed that LPCA has successfully suppressed irrelevant background features while enhancing the response to subtle lesion areas. This has directly translated to improved detection of early-stage DR features, minimizing both false positives and false negatives.

The ablation analysis has confirmed that the pixel-attention mechanism introduced by the LPCA module is crucial for the model’s superior performance. The spatial branch has strengthened lesion localization, while the channel branch has emphasized pathology-specific representations across multiple scales. Together, they have provided better generalization, higher sensitivity for early disease stages, and enhanced interpretability through clinically consistent activation maps.

### 4.8. Statistical Robustness and Clinical Sensitivity Analysis

To ensure the statistical reliability and clinical robustness of the proposed PAW-Net framework, additional analyses have been performed to assess model stability, the significance of performance improvements, and diagnostic sensitivity across different disease stages. The model has been trained and evaluated over five independent runs using varied random seeds and dataset partitions. Table 6 presents the mean ± standard deviation and 95% confidence intervals for key performance metrics accuracy, F1-score, and AUC. The narrow confidence intervals (<0.3%) indicate minimal variability across runs, confirming the reproducibility and stability of the proposed architecture. These results validate that the observed performance is consistent and not an artifact of random initialization or data partitioning.

Furthermore, to verify statistical significance, paired *t*-tests have been conducted across the five cross-validation folds, as shown in Table 7. As PAW-Net has the only classification model employed, intra-fold performance comparisons have been used to assess consistency and rule out random effects. The resulting *p*-values (<0.05) confirm that the variations across folds are statistically significant, reflecting stable learning behaviour and non-random performance gains. This analysis establishes that the improvements achieved by the proposed architecture are both quantitatively meaningful and statistically reliable.

In addition, a stage-wise sensitivity analysis has been conducted to evaluate the model’s clinical reliability in detecting lesions across different severities of DR. As shown in Table 8, the framework consistently achieved high sensitivity (>97%) across all stages, with 97.8% sensitivity for mild DR, the most clinically critical stage for early screening and intervention. These findings demonstrate that PAW-Net generalizes effectively across disease severities while maintaining strong detection performance during the early pathological phase.

The results confirm that PAW-Net exhibits high statistical reliability, strong reproducibility, and consistent clinical performance across independent runs and disease severities. The low variance, statistically significant *p*-values, and stable sensitivity across DR grades indicate that the model’s performance reflects genuine generalization rather than overfitting. These findings substantiate the robustness and clinical applicability of PAW-Net for large-scale, automated DR screening.

### 4.9. Comparison with State-of-the-Art Techniques

The performance of the proposed PAW-Net framework has been compared with several state-of-the-art models for DR detection and grading, as shown in Table 9. The PAW-Net model achieved an overall accuracy of 98.65% and an F1-score of 98.83%, outperforming all existing approaches. The integration of the LPCA module and the dual-decoder W-shaped architecture enhanced lesion localization precision and improved generalization across diverse datasets, thereby ensuring robust performance in 5-class DR classification. In comparison, Bappi et al. (2025) [14] achieved accuracies of 96.42% (Messidor) and 96.51% (APTOS) using a CNN framework with tailored preprocessing and class balancing. Although effective, their model underperformed by nearly 2% in both accuracy and F1-score compared to PAW-Net due to the absence of attention-driven mechanisms. Similarly, Qummar et al. (2024) [28] employed an ensemble of deep CNN models and achieved 80.8% accuracy, but the F1-score remained low (53.74%), indicating limitations in inter-class discrimination and generalization.

Cao et al. (2024) [29] implemented conventional machine learning algorithms (SVM, Neural Network, Random Forest) on the DIARETDB1 dataset and achieved strong binary classification accuracy (SVM = 98.5%). However, these classical methods lacked scalability for multi-class DR grading tasks, which PAW-Net effectively addressed through its multi-branch learning design. Likewise, Dai et al. (2024) [30] reported an accuracy of 96.1% and an F1-score of 93.4% using an AlexNet-based CNN for microaneurysm detection, but their single-lesion approach limited clinical applicability for holistic DR assessment. Menaouer et al. (2025) [31] adopted a hybrid model combining CNN, VGG16, and VGG19 on the Kaggle dataset, achieving 90.6% accuracy and an F1-score of 94%. Despite satisfactory results, their model suffered from reduced consistency across classes and datasets. In another study, Qureshi et al. (2025) [32] proposed an active deep learning CNN for EyePACS, reporting 98% accuracy and 93% F1-score; however, it failed to handle class imbalance and cross-dataset variability as effectively as PAW-Net. Jabbar et al. (2025) [33] used transfer learning with VGGNet on IDRiD and Messidor datasets and achieved 97.6% accuracy with an F1-score of 98.7%, indicating competitive performance, though slightly below PAW-Net’s generalization capability. Overall, the proposed PAW-Net exhibited the highest accuracy and F1-score among all compared methods. The LPCA-guided lesion refinement and dual-decoder architecture contributed significantly to its superior lesion interpretability, dataset adaptability, and clinical relevance.

While prior models such as CNN, ensemble, or transfer learning frameworks demonstrated strong single-dataset performance, they often struggled with multi-class grading stability and inter-dataset robustness. In contrast, PAW-Net consistently maintained high performance across DDR, Messidor + EyePACS, and APTOS datasets, underscoring its potential as a clinically reliable and scalable framework for automated DR detection and severity staging.

### 4.10. Limitations and Future Work

The proposed PAW-Net framework demonstrates strong performance in lesion segmentation and DR severity grading; however, several limitations persist. The model’s computational complexity arising from its dual-branch W-shaped architecture and LPCA module, demands substantial GPU memory and processing power, particularly for high-resolution fundus images. These requirements may create computational bottlenecks and restrict deployment in resource-limited or remote screening settings. Future efforts should therefore focus on architectural optimization to reduce computational load and memory consumption while maintaining diagnostic precision.

Additionally, interpretability for small lesion categories (e.g., exudates) remains challenging, where pixel-level attention may not fully capture subtle contextual cues. Enhancements such as multi-scale lesion priors, hybrid transformer-based attention layers, and clinical priors (e.g., optic disc-centred cropping and vessel-aware refinement) could address this limitation. Broader external validation across diverse clinical datasets is also essential to establish the model’s generalizability and clinical readiness.

In alignment with recent studies [34], future work could extend PAW-Net to explore retinal biomarkers as non-invasive indicators of systemic diabetic complications, such as nephropathy and neuropathy, thereby expanding its diagnostic relevance beyond ocular disease.

From a clinical integration standpoint, PAW-Net can be adapted for real-world ophthalmology workflows through:

(i) EMR-Linked Clinical Systems: As an AI-assisted triage and decision-support tool, PAW-Net can automatically analyze fundus images, generate severity scores, and store lesion overlays in patient EMRs to enable longitudinal tracking of disease progression and streamlined clinician review.

(ii) Cloud-Based Screening and AI-Assisted Reporting: A cloud-hosted deployment can support large-scale screening programs, allowing parallel image processing and integration with existing healthcare systems via Fast Healthcare Interoperability Resources (FHIR) standards. This would ensure scalable, interoperable, and accessible screening, even in under-resourced environments.

## 5. Conclusions

This study introduces PAW-Net, a novel pixel-attention-based W-shaped architecture designed for automated DR classification. Trained on DDR and Messidor + EyePACS and independently tested on APTOS 2019, the framework achieved an overall accuracy of 98.65%, with consistently high values for specificity, recall, precision, Dice coefficient, and F1-score, all exceeding 98%. The convergence of training and validation curves, combined with strong confusion matrix results, confirms that the model achieves balanced learning, minimizes overfitting, and generalizes effectively across datasets. Beyond numerical performance, PAW-Net distinguishes itself through its interpretability. The integration of lesion and vessel decoders enables the generation of segmentation masks, density maps, and radar-based biomarker visualizations that align closely with established clinical hallmarks of DR. These outputs provide both quantitative biomarkers and intuitive visual aids, enhancing trust and usability for ophthalmologists.

By simultaneously ensuring high accuracy, balanced performance across severity grades, and clinically meaningful interpretability, PAW-Net addresses key limitations of existing black-box deep learning systems. The findings confirm its potential as a reliable decision-support tool for large-scale DR screening and disease staging. Future work may focus on expanding its validation to real-world clinical settings and exploring its adaptability to other retinal diseases, thereby broadening its clinical impact.

## Figures and Tables

**Table 1:** Mathematical formulation of proposed framework.

Equation	Explanation	Variable Definitions
Input Transformation X = 𝒯(I)	Preprocessing pipeline applied to raw retinal image I, producing standardized input X.	I ∈ ℝ^H_0_×W_0_×3^: raw fundus image. X ∈ ℝ^H×W×3^: preprocessed image. 𝒯(·): denoising, resizing, cropping, normalization.
Lesion Prior Maps P = stack(P_1_, P_2_,…,P_k_)	Extraction of potential lesion structures using classical image filters, stacked into a multi-channel prior tensor.	P_k_ ∈ [0, 1]^H×W^: lesion prior for class k. P ∈ ℝ^H×W×K^: concatenated prior maps. K: number of lesion categories.
Encoder Representation F^ℓ^ = E^ℓ^ (F^(ℓ−1)^), F^(0)^ = X	Multi-scale encoder extracts hierarchical feature maps.	F^ℓ^ ∈ ℝ^H_ℓ_×W_ℓ_×C_ℓ_^: feature map at layer ℓ. E^(ℓ)^(·): convolution + pooling block. C_ℓ_: number of channels.
LPCA Fusion Φ = σ(W_a_[F^(ℓ)^||P]) ⊙ (softmax (QK^T^/√d) V) + (1 − σ(W_a_ [F^(ℓ)^||P])) ⊙ F^(ℓ)^	LPCA fuses encoder features with lesion priors, emphasizing pathology-rich regions.	Q = W_q_F^(ℓ)^, K = W_k_P, V = W_v_F^(ℓ)^: query, key, value projections. W_a_, W_q_, W_k_, W_v_: learnable weight matrices. ||: concatenation. σ(·): sigmoid activation. d: feature dimension. Φ: refined feature map.
Segmentation Decoder Ŝ_k_ = σ (Z_k_), Z_k_ = $D_{s}^{\left(k\right)}$ (Φ)	Decoder reconstructs lesion segmentation maps.	Ŝ_k_ ∈ [0,1]^H×W^: predicted probability map for lesion class k. $D_{s}^{\left(k\right)}$(·): deconvolution block. Z_k_: logit map.
Classification Branch $\hat{p}$ = softmax (W_c_h + b_c_)	Fully connected classifier grades DR severity levels.	$\hat{p}$ ∈ ℝ^5^: probability distribution. h = GAP(Φ): global average pooled feature vector. W_c_, b_c_: classifier weights and bias.
Pixel Attention Module F_pa_ = σ(W_p_* F^(ℓ)^) ⊙ F^(ℓ)^	Pixel-wise attention enhances salient regions in feature maps.	W_p_: convolutional kernel. *: convolution. F_pa_: pixel-attended feature representation.
Segmentation Loss L_seg_ = $\sum_{k=1}^{K}w_{k}\;(1-\frac{2({Ŝ}_{k},M_{k})+\varepsilon }{\left\|{Ŝ}_{k}{\|}_{1}+\right\|M_{k}{\|}_{1}+\varepsilon }$ + β $L_{BCE}^{\left(k\right)}$))	Hybrid Dice + Binary Cross-Entropy (BCE) loss ensures accurate lesion boundary segmentation.	M_k_: ground-truth mask for class k. Ŝ_k_: predicted segmentation map. w_k_: lesion-specific weight. ε: smoothing constant. β: BCE scaling factor.
Classification Loss Lcls=−∑c=04ωcyclogp^c	Weighted cross-entropy addresses class imbalance in DR severity grading.	y_c_: one-hot ground truth. ${\hat{p}}_{c}$: predicted probability for class c. ω_c_ = $\frac{N}{N_{c}}$ is class weight. *N*: total no. of training images *N_c_*: no. of images for class c.
L = λ_seg_ L_seg_ + λ_cls_ L_cls_ + λ_align_ L_align_ + λ_cons_ L_cons_	Final training objective balances segmentation, classification, alignment, and feature-consistency constraints.	λ_seg_ = 1.0, λ_cls_ = 1.0, λ_align_ = 0.5, λ_cons_ = 0.5: task-specific weights. L_align_: KL divergence aligning attention with priors. L_cons_: reconstruction consistency loss.

**Table 2:** Performance metrics.

Parameters	Equations
Precision (Psn)	$\frac{TP}{TP+FP}$
Sensitivity (Ssv)	$\frac{TP}{TP+FN}$
Accuracy (Acry)	$\frac{TP+TN}{TP+TN+FP+FN}$
Specificity (Scf)	$\frac{TN}{TN+FP}$
F1−Score	$2\times \frac{Psn\times Ssv}{Psn+Ssv}$
Dice Coefficient (Dic)	$2\times \frac{TP}{2TP+FP+FN}$

Where TP = True Positive, FP = False Positive, TN = True Negative and FN = False Negative.

**Table 3:** The description of Datasets.

Properties	DDR [24]	Messidor + EyePACS [25]	APTOS 2019 [26]
Total Images	12,522	1800	3662
No DR	6266	360	1805
Mild	630	360	370
Moderate	4477	360	999
Severe	236	360	193
Proliferative	913	360	295

**Table 4:** Hyperparameters used in PAW-Net.

Hyper-Parameter	Value
Initial Input Size	644 × 644
Activation Functions	ReLU, Leaky ReLU, Sigmoid
Number of Neurons in Each Convolutional Layer	75
Number of Hidden Layers	15
Decay	0.35
Momentum	0.5
Early Stopping	Yes
Batch Size	16
Dropout	0.75
Stride	2
Epochs	50
Learning Rate	0.001
Padding	Same
Optimization	Adam

**Table 5:** Ablation study results for the LPCA module in PAW-Net.

Model Variant	Accuracy (%)	F1-Score (%)	Dice (%)	AUC	Sensitivity (%)	Specificity (%)
Baseline U-Net	94.36	94.58	94.12	0.954	93.82	96.21
PAW-Net (without LPCA)	97.85	97.92	97.64	0.976	97.32	98.90
PAW-Net (LPCA–spatial-only)	98.10	98.20	98.03	0.979	98.00	98.90
PAW-Net (Full, with LPCA)	98.65	98.83	98.61	0.986	98.42	99.12

**Table 6:** Confidence Intervals for Performance Metrics.

Sr. No.	Metric	Mean	SD	95% Confidence Interval
1	Accuracy	98.65	±0.32	[98.41–98.89]
2	F1-score	98.80	±0.29	[98.58–99.02]
3	AUC	98.82	±0.26	[98.63–99.01]

**Table 7:** Statistical Significance Testing.

Sr. No.	Metric	t-Statistic	*p*-Value	Interpretation
1	Accuracy	4.32	0.021	Statistically significant
2	F1-score	4.75	0.018	Statistically significant
3	AUC	4.59	0.019	Statistically significant

**Table 8:** Sensitivity Analysis by Diabetic Retinopathy Stage.

Sr. No.	DR Stage	Sensitivity (%)	Precision (%)	AUC (%)
1	No DR	98.2	98.6	99.0
2	Mild	97.8	97.4	98.8
3	Moderate	98.6	98.7	98.9
4	Severe	99.1	99.3	99.2
5	Proliferative	99.0	99.2	99.3

**Table 9:** Comparison of outcomes with our model.

Sr. No.	Study	Dataset	Methodology	Analysis Type/Classes	Accuracy	Precision	F1-Score
1	Bappi et al. (2025) [14]	Messidor, APTOS-2019	CNN with tailored preprocessing & class balancing	5-class	Messidor = 96.42% APTOS = 96.51%	NA	Messidor = 96.95%, APTOS = 96.65%
2	Qummar et al. [28]	KAGGLE	Deep CNN models Ensemble	5-class	80.8%	63.85%	53.74%
3	Cao et al. [29]	DIARETDB1	1. SVM, 2. Single Hidden Layer NN, 3. Random Forest	Microaneurysm, Non-Microaneurysm	SVM: 98.5%, NN: 97.9%, RF: 97.2%;	NA	SVM: 92.6%, NN: 89.4%, RF: 88.8%
4	Dai et al. [30]	DIARETDB1	AlexNet CNN	Microaneurysm	96.1%	97.9%	93.4%
5	Menaouer et al. [31]	Kaggle	Hybrid Models (CNN, VGG16, VGG19)	5-class	90.60%	94.66%	94%
6	Qureshi et al. [32]	EyePACS	Active deep learning CNN Architecture	5-class	98%	N/A	93%
7	Jabbar et al. [33]	IDRiD and MESSIDOR	VGGNet and transfer learning	5-class	97.6%	NA	98.7%
Ours	PAW-Net	Train: DDR, Messidor + EyePACS; Test: APTOS-2019	Lesion-prior cross-attention + dual decoders	5-class	98.65%	98.47%	98.83%

## Data Availability

All datasets utilized in this study including DDR, Messidor + EyePACS, and APTOS 2019, are publicly available and have been appropriately cited in the manuscript references. The source code and training scripts developed for the proposed PAW-Net framework are available from the corresponding author upon reasonable request for academic and research purposes.
